# Iron deficiency markers in patients undergoing iron replacement therapy: a 9-year retrospective real-world evidence study using healthcare databases

**DOI:** 10.1038/s41598-020-72057-9

**Published:** 2020-09-11

**Authors:** Patrice Cacoub, Gael Nicolas, Katell Peoc’h

**Affiliations:** 1 Inflammation-Immunopathology-Biotherapy Department (DHU i2B), UMR 7211, UPMC Université Paris 06, Sorbonne Université, 75005 Paris, France; 2 UMR_S 959, INSERM, 75013 Paris, France; 3 CNRS, RE3632, 75005 Paris, France; 4 Department of Internal Medicine and Clinical Immunology, AP-HP, Groupe Hospitalier Pitié-Salpêtrière, 43-83 Boulevard de l’Hôpital, 75013 Paris, France; 5 Université de Paris, UFR de Médecine Xavier Bichat, Centre de Recherche Sur L’Inflammation (CRI), INSERM UMRs 1149, 75018 Paris, France; 6 Laboratory of Excellence, GR-Ex, Paris, France; 7 APHP, HUPNVS, Laboratoire de Biochimie Clinique, Hôpital Beaujon, 91118 Clichy, France; 8 Hôpital La Pitié Salpêtrière, 83 Boulevard de l’Hôpital, 75651 Paris, Cedex 13, France

**Keywords:** Biochemistry, Iron, Diagnostic markers

## Abstract

The diagnosis and treatment of iron deficiency is a primary public health goal. This study aimed to make an inventory of the use of biomarkers to assess the iron supply in patients given iron replacement therapy. A retrospective longitudinal real-world study of a cohort of patients receiving iron replacement therapy was conducted using data from healthcare coverage databases between January 2006 and December 2015 in France. The frequency of oral or intravenous iron treatment episodes preceded and/or followed by a biological assessment of iron deficiency was described. We then differentiate patients with or without chronic inflammatory diseases, which could impact the prescription. The evolution between 2006 and 2015 was also studied. The 96,724 patients received an average of 4.9 administrations of iron per patient, corresponding to 1.7 treatment episodes. In one-third of treatment episodes (34.6%), patients had a pre-treatment biological assessment, 15.5% a post-treatment assessment, and 7.3% both. The post-treatment measure of iron supply markers (i.e., Ferritin and transferrin saturation) was more frequent in patients suffering from chronic inflammatory diseases than in those without underlying chronic condition (22.6% to 41.0% vs. 3.1%; p < 0.0001). Serum ferritin was measured 30 times more than transferrin saturation measurements. The use of both tests increased steadily during the study period, although remaining low. Despite the recommendations, biological assessments of iron status are seldom prescribed and/or performed in the context of a pre- or post-treatment assessment, although more frequently realized in patients with chronic inflammatory diseases.

## Introduction

Iron deficiency is the most common and widespread nutritional deficiency in the world, with more than 1.2 billion affected individuals with anemia, and probably more than double without anemia^1,2^. Iron deficiency is defined by the presence of an insufficient supply of iron to meet the requirements of the organism; it can occur with or without anemia, which is characterized by the presence of a decrease in hemoglobin concentration and associated with microcytic hypochromic red cells^2^. Iron is crucial to numerous biologic functions, including cellular respiration, energy production, DNA synthesis, heme synthesis in erythroid cells and cell proliferation^3^. Iron deficiency, with or without anemia, is associated with fatigue, a negative impact on the life’s quality, decrease of productivity^4–6^, and delayed growth and development in children^7,8^. Iron deficiency is associated with higher mortality in certain chronic conditions including heart failure, kidney failure. Similarly, the presence of iron deficiency in patients undergoing heart surgery increases the risk of mortality at 90 days by a factor of 3.5, regardless of the presence or absence of anemia^9–12^. Iron deficiency is a significant public health problem, usually undiagnosed and untreated, and it is the cause of more than 60% of anemia worldwide. All-cause anemia is associated with increased morbidity and mortality in patients affected by inflammatory gastrointestinal diseases, cancer or kidney failure, and during and after surgery^13,14^. Despite the high prevalence and the potential risk of anemia which may ensue, iron deficiency remains under-diagnosed due to the absence of agreed and harmonized diagnostic test thresholds^15^ and/or the existence of a concomitant inflammatory context^4^. Indeed, when iron deficiency is associated with inflammation, the interpretation of biomarkers usually becomes less clear^16,17^. Two clinical-biological profiles have been defined from the pathophysiology—absolute iron deficiency and functional iron deficiency. Absolute iron deficiency, defined as a decrease in the total iron supply in the body, is caused by an insufficient iron intake or chronic blood loss. Absolute iron deficiency is defined by a low serum ferritin concentration and a low transferrin saturation (TSAT) index. Functional iron deficiency, due to a defect in iron transport from the storage areas, is principally linked to a persistent inflammatory state, together with the inappropriate increase of hepcidin^18^. Hepcidin, a peptidic hormone synthesized primarily in the liver, adjusts the plasma iron concentration which comes from the diet after absorption by the enterocytes of the duodenum, and from the iron recycling of the heme molecule by the macrophages. In both cases, hepcidin works by inducing the degradation of ferroportin, the only known iron exporter that displaces iron from an intracellular medium to an extracellular medium. Functional iron deficiency is characterized by a normal-to-high serum ferritin concentration and a low TSAT^9,19–21^. In France, according to the 2011 recommendations of the French National Authority for Health (Haute Autorité de Santé), the diagnosis of iron deficiency in the general population is based on serum ferritin concentration assessment. In chronic inflammatory diseases (including chronic heart failures [CHF], chronic kidney diseases [CKD]^22^, inflammatory bowel diseases [IBD]), ferritin measurement must be associated with the TSAT^23^, since inflammation leads to a functional iron deficiency.

The purpose of this study was to analyze retrospectively, using healthcare coverage databases, the use of the two primary markers of iron deficiency, *i.e.*, serum ferritin and TSAT, in patients given iron replacement therapy in France. The analysis covers a period between January 2006 and December 2015 and refers to a sample population of approximately 100 000 patients. We analyzed the frequency of iron-replacement treatment episodes administered orally or intravenously, preceded or not by a biological assessment of iron deficiency. The analysis was then stratified according to the presence of a chronic pathology. We also describe the temporal trends in the realisation of biological assessments.

## Methods

We retrospectively studied a longitudinal cohort of patients having received iron replacement therapy between January 2006 and December 2015. Patients from French healthcare coverage databases (Echantillon généraliste des bénéficiaires de l’assurance maladie [EGB])^24^, aged at least 18 years and with at least one iron deficiency treatment were included and followed until December 31, 2016. We retrospectively collected data regarding the main demographic and clinical characteristics of the patients, their pathologies, the measurement of biological markers of iron deficiency (ferritin and TSAT), and the corresponding iron deficiency treatments. This study adhere to RECORD guidelines^25^.

### Study population

Data used in this study were extracted from French healthcare databases, specifically the EGB, which is a random sample of 1/97 of the data from the national health database (Système National des Données de Santé [SNDS]) in France^24^. The SNDS, made available by the health insurance, is a source of medical and administrative data covering nearly 99% of the population of France. It includes reimbursement of medical costs follow-up (medical visits, treatments, laboratory and imaging tests, hospitalizations, and the presence of long-lasting illness [ALD]). The list of reimbursements for healthcare services was available for all individuals affiliated to the healthcare system since 2006, with a progressive integration of data from other insurance plans. The SNDS also includes data from the PMSI (Programme de Médicalisation des Systèmes d’Information), a database containing information on all medical diagnoses made during hospitalizations of short and medium duration (whether or not the diagnoses were the reason for hospitalization). These data were available for the period spanning January 2006 to the end of December 2015. The use of the EGB is regulated, and data were pseudo-anonymized. The sample taken for this study included all patients in the EGB treated for iron deficiency by iron administered orally or intravenously between January 2006 and December 2015. The study, therefore, is focused on prevalence and incidence cases occurring during this period. The date of the final data point for a patient is the date of death, exit from the EGB, or last available information (December 31, 2016). Patients aged below 18 years were not included in the study. Likewise, patients hospitalized for hemochromatosis or phlebotomy therapy were excluded from the present study.

### Variables

The records of orally or intravenously administration of iron occurring during the study period from patients aged above 18 years were extracted from the list of healthcare services in the EGB using CIP13 (Code Identifiant de Présentation) drug codes (Supplementary Table 1). For oral iron administrations, a new episode was defined by an administration occurring at least six months after the previous one and six months before the next (if the previous administration was unique). For intravenous (i.v.) iron administration, a new episode was defined as an administration occurring more than two months after the previous one; a single treatment episode could consist of one or two iron injections.

Ferritin and/or TSAT measurements performed for these patients were extracted from the biological table of the EGB. The ferritin and TSAT measurements were considered part of the pre-treatment assessment of iron deficiency when conducted less than three months from the first iron administration date for each episode. They were considered part of the post-treatment assessment of iron deficiency when performed less than three months from the last iron administration date for that episode.

Patients having received at least one iron treatment could be identified, and the demographic and clinical characteristics collected. Data for the following chronic pathologies were collected using ALD codes (long-lasting diseases : chronic heart failure (ALD5); chronic kidney disease (ALD19); inflammatory bowel disease (ALD24); cancer (ALD30); other chronic illnesses [peripheral arterial disease (ALD3), diabetes (ALD8), chronic respiratory failure (ALD14), cystic fibrosis (ALD18), vasculitis (ALD21), rheumatoid arthritis (ALD22), and spondylo-arthropathy (ALD27)].

### Statistics

The collected variables were described using standard statistical methods (mean, median, standard deviation, minimum and maximum values for continuous variables, and the number and percentage for categorical variables) and their 95% confidence interval. The percentages for iron therapy treatment episodes (oral or i.v.) preceded and/or followed by an assessment of iron deficiency markers (ferritin and/or TSAT) were described overall and by subcategory of chronic illness. For each chronic pathology considered, the percentage of episodes preceded and/or followed by an assessment of iron status markers in affected patients with the corresponding chronic condition was compared with non-affected patients using the chi-squared test.

The percentages of biological assessments of iron deficiency markers performed were estimated by calculating the number of ferritin and TSAT assessments per person-years. This analysis was performed for individuals affiliated with the general healthcare system (87% of subjects) because this system was the sole sampled for the entire study period. The analysis was performed on the secure portal of the national health insurance fund (Caisse Nationale d’Assurance Maladie) using the statistical software SAS Enterprise Guide version 4.3 (SAS Institute Inc., Cary, NC, USA).

## Results

### Sample description

Between January 2006 and December 2015, among the 817,140 persons included in the EGB, we excluded 16,207 persons aged below 18 years in 2016 and 750 persons diagnosed with hemochromatosis, resulting in a total population of 800,183. Consequently, our sample population consisted of 96,950 patients with at least one iron treatment between 2006 and 2015 (corresponding to 12.1% [96,950/800,183] of people included in the EGB during the same period). No intravenous administration of iron was coded in the PMSI databases (Programme de Médicalisation des Systèmes d’Information) for medicine-surgery-obstetrics and home hospitalization. The 96,950 patients having received at least one iron replacement therapy and included in the sample were mainly female (83.6%) with an average age of 46 years (Table 1). Compared to adults without iron treatment in the EGB, the proportion of females was higher in the iron deficiency treated population (83.6% versus 45.5%, p < 0.0001). The most frequent chronic diseases were IBD (0.7% versus 0.3%, p < 0.0001) and CKD (1.2% versus 0.4%, p < 0.0001).

**Table 1:** Main characteristics of the studied population (n = 96,950)—from French healthcare coverage databases (EGB) 2006–2013.

	n	(%)
**Gender**		
Men	15 872	(16.37)
Women	81 077	(83.63)
**Age**—Mean (SD)	46.1 (21.1)	-
**Chronic diseases**		
Cancer	9,391	(9.69)
Diabetes	7,454	(7.69)
Chronic heart failure	4,713	(4.86)
Peripheral arterial disease	2,253	(2.32)
Severe respiratory insufficiency	1687	(1.74)
Renal failure	1,160	(1.20)
Rheumatoid arthritis	905	(0.93)
Liver disease/cirrhosis	868	(0.90)
Inflammatory bowel disease	686	(0.71)
Vasculitis, lupus erythematosus, scleroderma	471	(0.49)
Spondyloarthritis	359	(0.37)

### Treatments for iron deficiency

Among the 96,950 patients under scrutiny, 96,724 (99.8%) were treated at least once with iron administered orally of which 96,175 (99.2%) were treated exclusively with iron administered orally and 549 patients (0,6%) received both oral and intravenous forms. Finally, 226 patients (0.2%) were treated exclusively intravenously.

The 96,724 patients received 478,941 administrations of iron during the study period (average of 4.9 administrations per patient) with a median of 3 (min: 1—max: 140) oral administrations per patient. These 478,941 iron administrations corresponded to 166,015 treatment episodes, which is a mean of 1.7 treatment episodes per patient with a median of 1 episode (min: 1—max: 13). The treatment episodes by orally-administered iron represented 99.8% of the total episodes in 96,724 patients with a median of 1 episode (min: 1—max: 12), and a median episode duration of 79.1 days (min: 1—max: 3,996). The median duration of oral treatment episodes was 79.6 days.

The number of patients receiving iron replacement treatment intravenously was 775 (0.8%; identified in the EGB between 2009 and 2015), for a total of 1,266 treatment episodes with a median of 1 episode (min: 1—max: 13). Compared with patients receiving iron treatment orally, those treated intravenously were more frequently men (37.5% versus 16.3%, p < 0.0001), older (56.8 years versus 46.1 years, p < 0.0001) and more often presented with a confirmed chronic illness declared in the ALD database (51.6% versus 30.6%, p < 0.0001).”

### Biomarkers of iron deficiency in patients receiving iron replacement therapy

Overall, approximately a third of the iron replacement treatment episodes were preceded by a biological assessment of iron status (pre-treatment assessment), consisting of the ferritin and/or TSAT measurements (34.6% for all episodes and 34.5% of the oral treatment episodes) (Table 2). The percentage of iron replacement therapy episodes followed by a biological assessment of iron status (post-treatment assessment) was approximately half of those episodes preceded by a biological assessment (15.5% for all episodes and 15.2% of oral treatment episodes). A minority of iron replacement treatment episodes were both preceded and followed by a biological assessment of iron stores (7.3% of all iron replacement therapy treatment episodes and 7.1% of oral treatment episodes). Among the biological assessments, serum ferritin concentrations were more frequently assayed than TSAT (about 30-fold), whether before or after iron replacement therapy treatment.

**Table 2:** Iron replacement treatment episodes preceded and/or followed by a biological assessment [transferrin saturation index and/or serum ferritin]**—**from French healthcare coverage databases (EGB) 2006–2013.

	Assessment before treatment		Assessment after treatment		Assessment before AND after treatment	
	n	(%)	n	(%)	n	(%)
**All**—**total sample (n = 166 015 episodes)**						
Transferrin saturation (TSAT)	1677	(1.0)	863	(0,5)		
Ferritin	56,081	(33.8)	24,952	(15.0)		
TSAT or ferritin	57,510	(34.6)	25,654	(15.5)	12,161	(7.3)
**Oral iron replacement therapy**—**total sample (n = 165 100 episodes)**						
TSAT	1631	(1.0)	823	(0.5)		
Ferritin	55,472	(33.6)	24,398	(14.8)		
TSAT or ferritin	56,890	(34.5)	25,087	(15.2)	11,661	(7.1)
**Iron replacement therapy—patients without chronic diseases (n = 126 755 episodes)**						
TSAT	978	(0.8)	433	(0.3)		
Ferritin	31,531	(24.9)	14,081	(11.1)		
TSAT or ferritin	43,881	(34.6)	16,607	(13.1)	7,774	6.1
**Iron replacement therapy—patients with chronic diseases (n = 29 171 episodes)**						
TSAT	544	(1.9)	351	(1.2)		
Ferritin	9,309	(31.9)	6,685	(22.9)		
TSAT or ferritin	10,107	(34.6)	6,948	(23.8)	3,438	(11.8)

**Figure 1:**
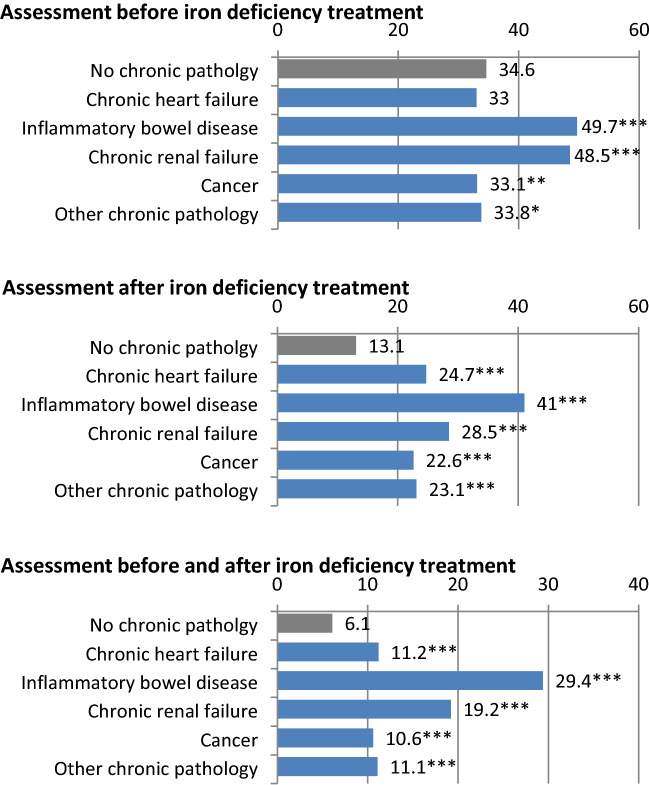
Iron therapy treatment episodes (%) preceded and/or followed by a biological assessment [transferrin saturation index and/or serum ferritin] according to the presence of chronic disease—from French healthcare coverage databases (EGB) 2006–2016. * p < 0.05 ** p < 0.01 *** p < 0,0001. IBD, inflammatory bowel disease. For each chronic pathology sub-group, the comparison was made with the group of iron deficiency treatment in patients without a chronic pathology (grey).

We also performed an in-depth analysis of patients presenting with chronic illnesses (Table 3, Fig. 1). The percentage of iron replacement treatments preceded by a pre-treatment assessment was higher for patients with IBD and CKD (49.7% and 48.5%, respectively) than those with no chronic inflammatory illness (34.6%; p < 0.0001). Post-treatment biological assessments were more frequently performed in patients with chronic diseases, all pathologies considered (range 22.6 to 41.0%) compared with patients with no chronic disease (13.1%, p < 0.0001). The most significant difference was observed in patients with IBD with a frequency of post-therapeutic biological assessment three times that of patients with no chronic diseases. Similarly, the proportion of iron replacement therapy treatments preceded and followed by a biological assessment was higher in patients with chronic diseases, all pathologies considered (range 10.6 to 29.4%), compared with patients with no chronic diseases (6.1%, p < 0.0001), with a marked difference for patients with IBD. Nevertheless, even for patients with IBD, less than half of the iron replacement episodes were preceded and/or followed by a biological assessment.

**Table 3:** Iron replacement treatment episodes preceded and/or followed by a biological assessment [transferrin saturation index and/or serum ferritin]**—**from French healthcare coverage databases (EGB) 2006–2013, detail of patients with chronic inflammatory diseases.

	Assessment before treatment		Assessment after treatment		Assessment before AND after treatment	
	n	(%)	n	(%)	n	(%)
**Iron replacement therapy**—**patients with chronic heart failure (n = 5,244 episodes)**						
Transferrin saturation (TSAT)	116	(2.2)	70	(1.3)		
Ferritin	1524	(29.1)	1,237	(23.6)		
TSAT or ferritin	1728	(33.0)	1,296	(24.7)	589	(11.2)
**Iron replacement therapy**—**patients with inflammatory bowel disease (n = 2,548 episodes)**						
TSAT	51	(3.3)	40	(2.6)		
Ferritin	670	(43.5)	614	(39.8)		
TSAT or ferritin	766	(49.7)	632	(41.0)	453	(29.4)
**Iron replacement therapy—patients with chronic kidney disease (n = 1,034 episodes)**						
TSAT	29	(2.8)	16	(1.5)		
Ferritin	443	(42.8)	283	(27.4)		
TSAT or ferritin	501	(48.5)	295	(28.5)	199	(19.2)
**Iron replacement therapy—patients with cancer (n = 10 591 episodes)**						
TSAT	190	(1.8)	121	(1.1)		
Ferritin	3,103	(29.3)	2,300	(21.7)		
TSAT or ferritin	3,504	(33.1)	2,396	(22.6)	1,124	(10.6)
**Iron replacement therapy—patients with another chronic disease (n = 16 014 episodes)**						
TSAT	274	(1.7)	184	(1.1)		
Ferritin	5,199	(32.5)	3,570	(22.3)		
TSAT or ferritin	5,417	(33.8)	3,702	(23.1)	1779	(11.1)

**Figure 2:**
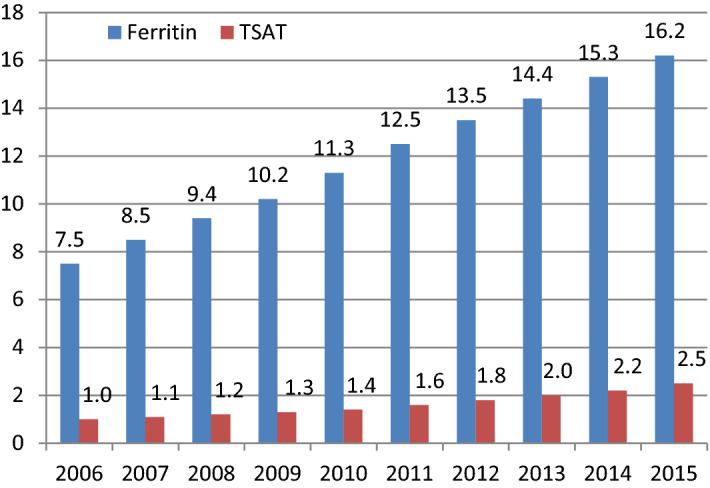
Annual frequency (%) of the biological assessments of iron deficiency (transferrin saturation index [TSAT] and/or serum ferritin)—from French healthcare coverage databases (EGB) 2006–2015.

The temporal analysis of the frequency of biological assessments of iron deficiency (ferritin and TSAT) performed using the EGB data for the study period showed a steady increase in the realization of these analyses over ten years (Fig. 2). For TSAT, the realisation increased from 1.0 to 2.5 per 100 person-years (+ 155%), and for serum ferritin, from 7.5 to 16.2 per 100 person-years (+ 116%). TSAT measurements were far fewer than the ferritin measurements, and the proportion represented by these two tests was constant throughout the study period.

## Discussion

Iron deficiency, associated or not with anaemia, is the most common nutritional deficiency in the general population. Despite its high prevalence, iron deficiency remains under-diagnosed and under-treated, even in high-income countries. The biomarkers recognized as most effective for both iron deficiency screening and treatment follow-up are serum ferritin and TSAT. The combined use of these tests increases their validity, particularly in the presence of chronic inflammatory diseases. Serum ferritin level is the most performant exam for the identification of iron deficiency (concentration < 30 microgr/L^26–28^, with variations according notably to the gender and ag^29^). Concentrations thresholds are different in some chronic inflammatory diseases, i.e. ferritin concentrations < 100 μg/L or < 300 μg/L^5^. A TSAT lower than 16% indicates that iron supply is insufficient to support normal erythropoiesis^30^.

To better understand the real-life management of iron deficiency in France, we analysed data from healthcare coverage databases for the percentage of iron replacement treatment episodes preceded and/or followed by a biological assessment of iron deficiency, overall and in the presence of chronic disease. The principal lessons learned from this study are (i) biological assessment of iron deficiency, serum ferritin and/or TSAT measurements preceded only 34% of iron treatment episodes, and was realised in 15% after treatment; (ii) these assessments were more frequently performed in patients with long-lasting chronic inflammatory diseases, but the rate at which they were performed remained considerably below expectations.

As for any deficiency, the management of iron deficiency proceeds in a series of essential steps, namely diagnosis using recommended biological markers and a follow-up of these markers during treatment. Although the efficacy of oral iron administration has been known for a long time, still there are many uncertainties on the better type of iron, on the dose^31^. Our study showed that in France, only a third of patients receiving iron replacement therapy had a specific pre-treatment biological assessment, and a biological assessment followed only one-seventh of all episodes. The frequency of biological testing was higher in patients with chronic illness at high-risk of iron deficiency: half of the patients presenting with CKD or IBD had a pre-treatment assessment. However, for other chronic inflammatory diseases, the results were also weak, especially for CHF and cancer. Chronic inflammation is often associated with iron deficiency^2,5,6^. Iron deficiency is found in 37% to 61% of patients with CHF, 24% to 85% of patients with CKD and 13% to 90% of patients with IBD^6,9,19,21,32^. Therefore, in most cases treatments were initiated without confirming the diagnosis and evaluating the severity of iron deficiency. Moreover, when patients deemed iron-deficient received iron replacement therapy, the impact of the treatment on the iron deficiency was not evaluated biologically^33^. The measure of TSAT was rarer still, even in patients with chronic inflammatory illness. However, TSAT is recognized as the gold standard for accurately diagnosing iron deficiency in chronic diseases^15,16^.

This study nevertheless shown a steady rise in the use of ferritin and TSAT between January 2006 and December 2016, evidencing an increasing awareness of the importance of diagnosis and follow-up during treatment for iron deficiency. During this same period, the proportion of ferritin measurements to TSAT remained constant, suggesting that these exams were not necessarily tailored to the pathophysiological context^33–35^.

This study has some limitations. The intravenous iron replacement treatments were not singled out as they were not coded as an input of the hospitalizations. Besides, they were removed from the over-the-counter provision in 2013 to subsequently being limited to administration during hospitalization. The median duration of oral treatment episodes was 79.6 days, to which an additional month should be added because the calculation corresponds to the time elapsed between the first and last administration for the considered treatment episode.

In conclusion, in real-life in France, the two most relevant biomarkers recognized in the diagnosis of iron deficiency, serum ferritin and TSAT, are rarely measured before and/or after iron replacement treatment episodes. Even in high-risk situations, such as chronic inflammatory diseases, the biological exploration of iron deficiency is remarkably low. This situation has to be improved since non-absorbed iron could exert some deleterious effects, particularly in IBD^36^. Substantial efforts to inform and train medical teams are mandatory to improve the care of patients presenting with iron deficiency.

## Supplementary information

Supplementary file1

## Data Availability

The data used was extracted from French healthcare databases, specifically the EGB, which is a random sample of 1/97 of the data from the national health database in France.

## Code availability

Data described in the manuscript, codebook, and analytic code will be made available upon request pending application and approval.
