# Supplementary material for: Iron deficiency markers in patients undergoing iron replacement therapy: a 9-year retrospective real-world evidence study using healthcare databases
Source: Sci Rep. 2020 Sep 11;10:14983. doi: 10.1038/s41598-020-72057-9 (PMC7486365; doi:10.1038/s41598-020-72057-9)
Supplement: Supplementary file 1 — Supplementary file1 [file 41598_2020_72057_MOESM1_ESM.docx]

Iron deficiency markers in patients undergoing iron replacement therapy: a 9-year retrospective real-world evidence study using healthcare databases

Patrice Cacoub, Gael Nicolas, Katell Peoc’h

Supplementary Table

Supplementary Table 1: Product name and drug code of available iron in France

| **Product name** | **Drug code** |
| --- | --- |
| TOT'HEMA sol in amp 20 50mg | 3400931073130 |
| ASCOFER 33 mg cap | 3400930069608 |
| FER ARROW 100 mg/5 ml inj sol | 3400957745806 |
| FER MYLAN 100 mg/5 ml dil sol for drip | 3400957403744 |
| FER PANPHARMA 100 mg/5 ml dil sol for drip | 3400958004780 |
| FER SANDOZ 100 mg/5 ml dil sol for drip | 3400957347352 |
| FERINJECT 50 mg/ml inj sol for drip 2 vials/10ml | 3400921939484 |
| FERINJECT 50 mg/ml inj sol for drip vial/10ml | 3400938692471 |
| FERINJECT 50 mg/ml inj sol for drip vial/20ml | 3400958598852 |
| FERINJECT 50 mg/ml inj sol for drip vial/2ml | 3400938681246 |
| FERO GRAD VITAMINE C 105mg CPR 30 | 3400931187943 |
| FERRIPROX 100 mg/ml sol bib | 3400939438542 |
| FERRIPROX 1000 mg EC | 3400949627769 |
| FERRIPROX 500 mg EC | 3400936576285 |
| FERRISAT 50 mg/ml inj sol for drip 2Amp/10ml | 3400957292201 |
| FERRISAT 50 mg/ml inj sol for drip 5Amp/10ml | 3400957292379 |
| FERRISAT 50 mg/ml inj sol for drip 5Amp/2ml | 3400957147969 |
| FUMAFER 33 mg/1 g oral powder | 3400930418154 |
| FUMAFER 66 mg EC 100 | 3400930418093 |
| FUMAFER 66 mg EC 50 | 3400955917441 |
| FUMAFER powder | 3400930418154 |
| INOFER 100 mg EC 33mg | 3400933529697 |
| MONOVER 100 mg/ml inj sol or for drip 2 vials/10ml | 3400955020424 |
| MONOVER 100 mg/ml inj sol or for drip 5 vials+stopper/1ml | 3400955020332 |
| MONOVER 100 mg/ml inj sol or for drip 5 vials+stopper/5ml | 3400955020400 |
| TARDYFERON 50 mg EC30 | 3400928025777 |
| TARDYFERON 80 mg EC | 3400957917043 |
| TARDYFERON 80 mg EC 30 | 3400933518004 |
| TARDYFERON B9 EC | 3400932918195 |
| TIMOFEROL 50 mg EC 30 | 3400927946424 |
| TIMOFEROL 50 mg EC 90 | 3400927946653 |
| VENOFER 20 mg/ml inj sol i.v. | 3400957128340 |
